# *GRASP* [Genomic Resource Access for Stoichioproteomics]: comparative explorations of the atomic content of 12 *Drosophila* proteomes

**DOI:** 10.1186/1471-2164-14-599

**Published:** 2013-09-04

**Authors:** James D J Gilbert, Claudia Acquisti, Holly M Martinson, James J Elser, Sudhir Kumar, William F Fagan

**Affiliations:** 1 A08 Heydon-Lawrence Bdg, University of Sydney, Sydney NSW 2006, Australia; 2 University of Maryland, College Park, MD 20742, USA; 3 WWU Munster, Institute for Evolution and Biodiversity, Hufferstr. 1, Munster 48149, Germany; 4 Center for Evolutionary Medicine and Informatics, Biodesign Institute, Arizona State University, Tempe, AZ 85287-5301, USA; 5 School of Life Sciences, Arizona State University, Tempe, AZ 85287-4501, USA

**Keywords:** Bioinformatics, Comparative-phylogenetic analysis, Ecological stoichiometry, Material costs, Nutrient limitation, Proteomics

## Abstract

**Background:**

“Stoichioproteomics” relates the elemental composition of proteins and proteomes to variation in the physiological and ecological environment. To help harness and explore the wealth of hypotheses made possible under this framework, we introduce *GRASP* (http://www.graspdb.net), a public bioinformatic knowledgebase containing information on the frequencies of 20 amino acids and atomic composition of their side chains. *GRASP* integrates comparative protein composition data with annotation data from multiple public databases. Currently, *GRASP* includes information on proteins of 12 sequenced *Drosophila* (fruit fly) proteomes, which will be expanded to include increasingly diverse organisms over time. In this paper we illustrate the potential of *GRASP* for testing stoichioproteomic hypotheses by conducting an exploratory investigation into the composition of 12 *Drosophila* proteomes, testing the prediction that protein atomic content is associated with species ecology and with protein expression levels.

**Results:**

Elements varied predictably along multivariate axes. Species were broadly similar, with the *D. willistoni* proteome a clear outlier. As expected, individual protein atomic content within proteomes was influenced by protein function and amino acid biochemistry. Evolution in elemental composition across the phylogeny followed less predictable patterns, but was associated with broad ecological variation in diet. Using expression data available for *D. melanogaster*, we found evidence consistent with selection for efficient usage of elements within the proteome: as expected, nitrogen content was reduced in highly expressed proteins in most tissues, most strongly in the gut, where nutrients are assimilated, and least strongly in the germline.

**Conclusions:**

The patterns identified here using *GRASP* provide a foundation on which to base future research into the evolution of atomic composition in *Drosophila* and other taxa.

## Background

Understanding the basis of biological diversity requires integration of ecological and evolutionary information. One exciting emerging picture is that ecological variation in the availability of key elements can have evolutionary consequences even at the primary protein sequence level [1-4], a perspective known as “stoichioproteomics” (reviewed in [5]). Indeed, various studies, both older and newer, have detected selection for efficiency of usage in key limiting elements in amino acid side chains, both in the sequences of individual proteins [1,6] and across entire proteomes [7-10].

To begin to explore this potentially vast source of variation within and among species, it is necessary to have reliable and comparable sequence datasets for multiple taxa. This problem applies most strikingly in multicellular eukaryotes. Although several studies have explored stoichioproteomics in prokaryotes (e.g., [7,8,11-15]) and eukaryotes [2,9-11], prokaryotic species have more often been the subject of comparative analysis than eukaryotes. Comparative analyses of molecular-scale variations in the elemental compositions of proteins among plant and animal species are currently very scarce (see e.g. [2,9]). One reason for this may be that*,* in these taxa, such analyses are more difficult owing to the more complicated relationships between gene, transcript, and protein (for example, through alternative splicing), which blurs the definition of “homology” and makes meaningful comparisons among proteomes more difficult to achieve. In such species, answering even simple questions about atomic composition can quickly become a daunting task that requires merging several large datasets from different research groups using multiple sequence identity codes.

To begin to address this problem, we present *GRASP* (Genomic Resource Access for Stoichioproteomics, URL: http://www.graspdb.net), a public web resource focused on providing a centralized and standardized resource for analyzing the elemental composition of whole eukaryotic proteomes. *GRASP* is intended, first and foremost, to encourage and enable researchers to conduct their own comparative stoichioproteomic analyses. Second, it is intended to simplify and greatly facilitate these analyses for eukaryotes, by providing a common, standardized repository of protein-by-protein information with easy ways to search, match, extract, and analyse composition data from groups of homologous proteins and splice variants across multiple species with sequenced genomes. Third, we seek to facilitate testing of biological hypotheses by linking protein data to other publicly available sources of biological information using standard naming conventions. *GRASP* does not provide new data; rather, the advance *GRASP* represents is one of convenience and streamlining of analyses that would otherwise be laborious, in a manner analogous to repositories of biological data such as FishBase (http://www.fishbase.org), the Tree of Life (http://www.tolweb.org) and the Global Biodiversity Information Facility (http://www.gbif.org).

In its current form, *GRASP* includes information on the atomic composition of proteins of all twelve fully-sequenced species of *Drosophila: D. ananassae, D. erecta, D. grimshawi, D. melanogaster, D. mojavensis, D. persimilis, D. pseudoobscura, D. sechellia, D. simulans, D. virilis, D. willistoni and D. yakuba.* Information on multiple splice variants is currently available for *D. melanogaster*. In the future, we plan to expand the database to include a diversity of multicellular and unicellular eukaryotes.

### Exploring *Drosophila* stoichioproteomics

Combined with an almost unparalleled understanding of the biology of *Drosophila* from many decades of intensive research (see [16] and references therein), these 12 sequenced genomes have already been used to make inferences about species relationships and speciation, patterns of genome organization, e.g. [17], the evolution and function of gene sequences, e.g., [18], and rates of evolution, e.g., [19]. However, the potential of this clade for studying variation in atomic composition has yet to be investigated.

To illustrate the potential that *GRASP* represents for researchers interested in testing biological and ecological hypotheses using stoichioproteomic data, and the kinds of analyses that are facilitated by *GRASP*, we present here the first exploratory analysis and overview of proteomic variation in atomic composition among the 12 sequenced *Drosophila* species. We specifically illustrate the potential of this resource by conducting preliminary tests of two stoichioproteomic hypotheses.

### Example hypotheses

Stoichioproteomics derives a number of specific hypotheses from a single core precept: that limitation in an element leads to purifying selection in order to reduce the usage of amino acids needing that element in protein sequences or their expression. Limitation could occur at any of several levels. At the long-term ecological level, limitation would result in predictable changes in protein stoichiometry among species (see [2]). Alternatively, at the short-term ecological level, limitation would result in predictable changes in protein stoichiometry among or even within individuals (see [20]). Limitation may operate even at the intracellular level, whereby temporary nutrient limitation within cells due to the demands of protein expression results in predictable changes in composition among proteins with predictable expression profiles such as nutrient assimilation proteins [1], differentially expressed protein variants [6], or according to transient expression profiles [13]. We examined two sets of predictions arising from this general elemental limitation hypothesis: first, ecological differences should lead to predictable protein stoichiometry among species, and second, highly-expressed proteins should have sequences conservative in key nutrients, specifically N.

First, we collated data for the 12 *Drosophila* species on key ecological traits that have been linked to organismal stoichiometry, and tested for associations between these traits and the composition of proteins (and protein subsets) across species. These traits, along with the relevant predictions with respect to *Drosophila* proteomes, are outlined in Table 1.

**Table 1:** Predictions about associations between genomic and ecological traits and the evolution of protein atomic content

**Trait(s)**	**Expected or potential associations with protein stoichiometry**
Genome size (130 – 364 Mb); Intron percent (19.6–24.0%) [17]	Larger proteomes (as indicated by larger genomes) require more intensive translation activity, and so should contain proportionally less of a limiting element (C, N or S), owing to selection for efficiency of element usage. This may be confined to the proteins involved in translation, which are overexpressed when intensive translation is required. Similarly, higher percentages of protein–coding DNA (i.e. lower intron percent) should select for higher efficiency of usage owing to a proportionally greater effect on the phenotype.
Male and female body size (thorax width; males, 0.64–1.78 mm; females, 0.80–1.89 mm); Sexual dimorphism (female thorax – male thorax; 0.00–0.18 mm); Development time (10–27d); Male and female specific growth rate (development time/thorax width) [16]	Contrasting predictions arise from the growth rate hypothesis [21]. First, smaller organisms have faster specific growth rates than larger organisms and therefore require proportionally more transcription activity. Thus they should require more nutrient conservation in proteins, particularly the proteins of transcription and translation that are overexpressed when increased protein synthesis is required. Conversely, owing to higher rates of protein synthesis, smaller organisms have a lower protein:nucleic acid ratio, the N:P ratio of their tissues is accordingly lower, and they are predicted to be more easily P limited rather than N limited. This predicts that smaller organisms should be under weaker selection to conserve nitrogen in their protein sequences.
Ovariole number (16–43) [16]	With limited nutritional choices, organisms often prioritize allocation to fertility over lifespan [22]; this may impose selection for nutrient conservation in key proteins. Therefore evolutionary increases in ovariole number should result in nutrient depletion across proteomes, or differentially in the proteins of oogenesis.
Diet breadth (general, oligo, specialist) [16], [25], [34]	It is advantageous to maintain a stoichiometric balance close to that of one’s food (reviewed in [23]). Generalist flies are more likely to be able to adjust the nutritional balance of ingested food to their own nutrient demands [24], whereas specialist flies are more likely to evolve a body stoichiometry that corresponds with that of their resources [25]. Therefore the evolution of feeding specialization may involve evolution of distinctive protein stoichiometry, across proteomes or in the proteins of nutrient assimilation and digestion.

Ranges of trait values are given in parentheses where appropriate.

Second, we combined the data in *GRASP* with a public database of protein expression (FlyAtlas, http://www.flyatlas.org[26]) in different *Drosophila* tissues to test for a negative association between protein expression and N content. Using tissue-specific expression allowed us to assess the predicted relationship not only in a general context but also in tissues where this relationship would be expected to apply strongly or not at all, respectively. First, the insect midgut is the site of nutrient uptake and assimilation; enzymes that function to assimilate nutrients have evolved to contain less of the element they assimilate [1], leading to the prediction that we should observe a stronger relationship between expression and N content in the midgut. Conversely, in the testes of *D. melanogaster*, evidence suggests that protein synthesis is greatly reduced, which should reduce the requirement for N conservation. Protein expression during spermatogenesis in *Drosophila* occurs in a unique way: transcription occurs only in early meiotic divisions, which peak during the pupal stage. Post-meiosis, there is almost no transcription in *Drosophila* spermatids; instead, protein synthesis is achieved by retention of mRNA transcripts for relatively long periods of time [27]. Translation also appears to be reduced—12 ribosomal proteins are down-regulated in adult testes while none is up-regulated [28]. In a global expression study, transcription and translation proteins were not among those differentially expressed in testes, unlike in ovaries [29]. Thus, in contrast to other tissues, an adult testis would have no particular requirement for N conservation in its proteins, because proteins are being synthesized at a much lower rate.

## Results and discussion

### Overview

In *GRASP*, core information on individual proteins is given as protein length, the percent composition of each amino acid, the number of atoms of each constituent element in each protein (excluding invariant backbone), and “elemental content”, defined (following [2]) as

$$\left(100/L_{\mathrm{protein}}\right).\sum w_{i}p_{i}$$

where *L*_*protein*_ is the protein sequence length, *w*_*i*_ is the number of atoms of a given element in the side chain of amino acid *i,* and *p*_*i*_ is the frequency of amino acid *i* in the protein.

The multivariate analyses we present here incorporate (1) elemental content, following previous authors, e.g. [1,2,7,10], (2) DNA GC content, and (3) several basic properties of amino acid sequences (protein length, proportions of hydrophobic, polar, positive, negative, and aromatic residues). We restricted our analyses to the subset of proteins that have orthologs in all 12 available *Drosophila* species (n = 4934). Future authors may wish to base more detailed analyses upon individual amino acid contents and raw numbers of constituent elements, or on the composition of proteins lost and gained during the evolution of this clade.

### Standing variation

**Figure 1:**
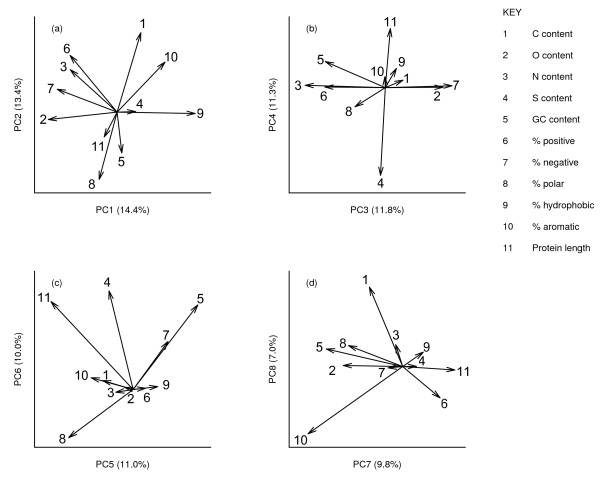
**a-d biplots showing variable loading vectors for the first eight principal component axes describing standing variation in protein composition for 12 combined *Drosophila *proteomes.**

Principal component analysis revealed multiple patterns of co-linearity among the analysed sequence variables (Figure 1).

**Figure 2:**
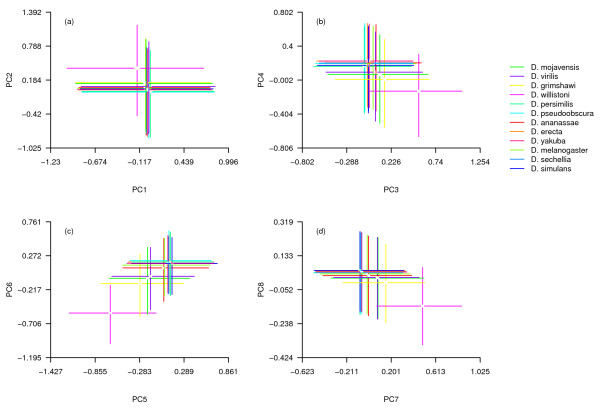
**a-d composition of 12 *Drosophila *proteomes (lines, interquartile range; central gaps, 95% confidence intervals around the median).** Axes are the same as in Figure 1.

**Figure 3:**
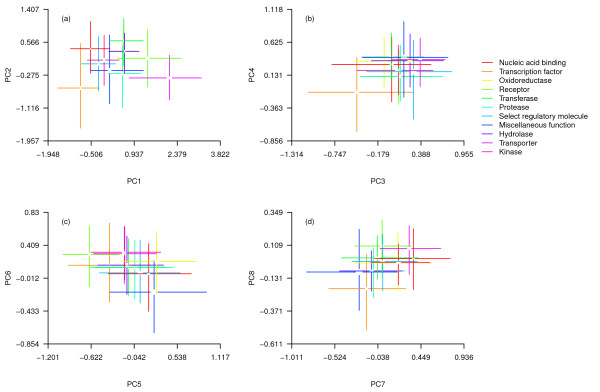
**a-d proteomic composition of different functional groups of proteins within 12 *Drosophila *proteomes (lines, interquartile range; central gaps, 95% confidence intervals around the median).** Axes are the same as in Figure 1.

Both species and functions differed statistically (MANOVA, species: Pillai’s trace = 0.91, df = 27, p < 0.001; function: Pillai’s trace = 0.29, p < 0.001) but the interaction of species and function did not (Pillai’s trace = 0.02, p = 0.99), indicating that protein functional groups occupied similar regions of multivariate space relative to each other within each species’ proteome. There were relatively small differences among species with the exception of *D. willistoni* (Figure 2) but pronounced differences among protein categories (Figure 3).

Figure 1 shows pairwise plots of variable loadings for the first eight principal component axes, which collectively explained 89% of the variance. Most of the co-linearity stemmed from inherent properties of protein sequences. Aside from fundamental associations such as those between N and O content and charge density (described by PC1), and between C content and aromaticity (described by PC2), Figure 1b also shows, for example, that S content was negatively associated with protein length. Although this partly reflects the effect of a constant initial methionine residue, PC1 and PC2 loadings did not appreciably change after excluding this initial residue from all proteins (data not shown) so this may stem from the tendency of smaller proteins to be stabilized by disulphide links while longer proteins tend to have salt bridges [30]. Also reflecting previous findings, DNA GC content was correlated negatively with protein C content [14,15] and also with O content [8].

Most species showed only small differences on all PC axes (Figure 2). *D. willistoni* was an outlier in many cases, notably PC2, PC3 and PC7, stemming from its proteome’s relatively exceptionally high O content (median 0.496 atoms/residue) and its genome’s well-documented low GC content (median for our dataset 46.5%; see [31]). Although *D. willistoni* is not exceptional among eukaryotes either in its GC or O content, since it falls roughly centrally among eukaryotes plotted in Vieira-Silva & Rocha's Figure 2 (in [8], p. 1935), it was a clear outlier within the clade studied here.

Overall, protein functional categories differed in elemental content and sequence properties largely in line with expectations from the biochemistry of each protein category (Figure 3). For example, transcription factors and nucleic acid binding proteins had very low values on PC1, indicating high N and O content, high charge density and hydrophilicity; nucleic acid binding proteins in particular had high N content, reflecting the requirement for positive charge associated with binding to negatively charged DNA. In contrast, receptors and transporters had high values of PC1 indicating very low N and O, low charge density, and high hydrophobicity, consistent with the high proportion of hydrophobic groups required to function within a plasma membrane.

### Patterns in elemental content across the whole phylogeny

**Figure 4:**
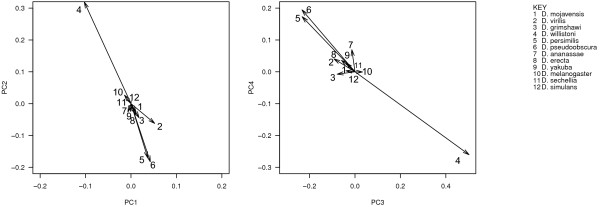
**Biplot showing mean divergence in proteomic composition since the most recent ancestor for 12 species of *Drosophila.*** Ancestral values are standardized at the origin for comparison. Axes are the same as in Figure 1.

To investigate evolutionary patterns rather than standing variation, we first explored species divergence in the PC axes identified above by reconstructing ancestral states for each axis in the PCA. The most striking lineage-specific patterns were in *D. willistoni* and in the *D. persimilis*/*D. pseudoobscura* lineage (Figure 4). *D. willistoni* has undergone across-the-board increases in PC2 (i.e. increased C content and decreased GC content, percentage of polar residues, and protein length) and PC3 (i.e. increased O content and negative charge, and decreased GC content, N content and positive charge) and decreases in PC4 (i.e. increased S, reduced protein length) in all functional categories of proteins, arising from divergence since its last ancestor, while *D. pseudoobscura* and *D. persimilis* have both undergone equally wholesale, but slightly less substantial, decreases in PC2 and PC3 and increases in PC4. This suggests a proteome-wide, bottom-up evolutionary pressure leading to correlated changes in GC content of DNA and of C, O and N content across proteins, instead of an ecological or physiological explanation that might be seen more strongly in some proteins than in others.

Why we should observe this pattern remains an open question. It seems likely that selection acting on DNA GC content may drive the observed difference; PC2, PC3 and PC4 all contain heavy loadings for GC content, a fundamental property of DNA, and, in PCA analyses, extant standing variation in species tended to fall along a common line roughly parallel with variation in GC content in all cases (Figure 2). While it is beyond the scope of this study to speculate on causal relationships between genomic GC content and protein properties, which are currently unclear (for discussion, see [8] with respect to O content and [15] with respect to C content), changes in GC content in *D. willistoni* have been shown to correlate with changes in amino acid transition rates [32]. If evolutionary changes in GC content indirectly drive evolution in amino acid composition and protein properties such as O content, it is likely that this change may be sufficient to account for observed differences in PC2, PC3 and PC4 between *D. willistoni*, *D. pseudoobscura*, *D. persimilis* and their congeners.

**Figure 5:**
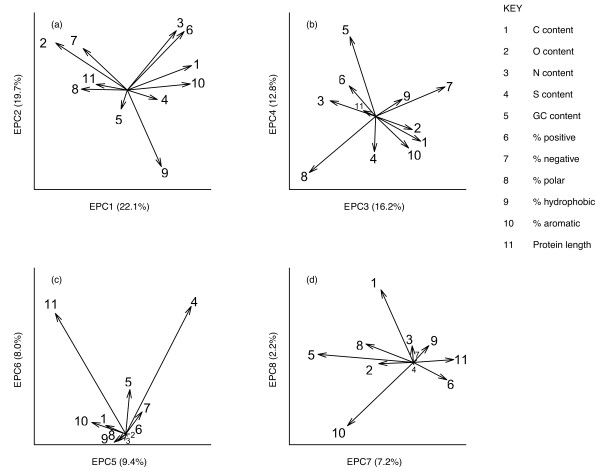
**a-d biplots showing variable loading vectors for the first eight evolutionary principal component (EPC) axes describing phylogenetically independent contrasts in protein composition across the *Drosophila *phylogeny, i.e. summarizing evolutionary changes in proteomic composition rather than extant standing variation.**

We then calculated phylogenetically independent contrasts in each variable for each ortholog set across the phylogeny to look at general patterns in the evolution of proteomic properties across the clade. We amalgamated all independent contrasts into a single dataset and used PCA to identify broad patterns of evolutionary variation (following [33]). The main axes of variation we identified are given in Figure 5. Here we term these “Evolutionary Principal Components” (EPC) purely to distinguish them from the axes identified for standing variation among proteomes in Figure 1. Broadly speaking, these were (EPC1) from long, polar, high-O content proteins to aromatic, high-C proteins; (EPC2) from hydrophobic, high-GC proteins to positive, high-N proteins, (EPC3) from more polar proteins to more negative, more hydrophobic proteins and (EPC4) from GC-poor, S-rich, aromatic proteins to GC-rich, positively charged proteins. Evolutionary changes in protein and DNA properties often mirrored patterns detected within proteomes. For example, O content and hydrophobicity were strongly opposed on both PC1 (Figure 1) and EPC1 (Figure 5). As another example, evolutionary divergence in both C and O content were negatively related to divergence in GC content on EPC3, which reflected our findings within proteomes (Figure 1) and supports previous findings among whole proteomes of other taxa [8,14,15].

However, evolutionary patterns were sometimes different from standing variation within proteomes. Evolution in N content and % positive charge followed patterns different from those seen in static variation. Evolutionary changes in N and % positive charge were independent of O content and % negative charge both on EPC1 and (to a lesser extent) on EPC3 (Figure 5). In contrast, within proteomes, these two variables were positively related to O content and % negative charge on PC1 (collectively describing charge density) and negatively related on PC3 (collectively describing a positive–negative continuum; see Figure 1).

### Testing hypotheses across the phylogeny

Statistics for all protein subsets are given in Table 2. Note that we use PC (not EPC) axes for these analyses, because the technique we used (PGLM) uses raw species values, not contrasts, as its input data (see Methods for details). We detected extensive changes in protein stoichiometry across all axes with intron percent; associations significant at the p < 0.01 level were seen in 651, 561, 493 and 539 out of 4934 proteins on PC1, 2, 3 and 4, respectively, over 10 times the expected number. However, there was no detectable bias on any of PC1-4 towards positive or negative associations with intron percent (binomial tests on effect sizes, all NS); neither were positive or negative associations biased towards any protein category (χ^2^ tests, all NS). This indicates an overall lack of consistent association between intron percent and protein stoichiometry. Comparably high numbers of significant associations that also had no net positive or negative bias were also detected for ovariole number (162–394 proteins across all axes) and specific development time (161–393 proteins across all axes). Future investigators may wish to explore these associations in more detail, on a protein-by-protein basis.

**Table 2:** **Number of proteins (out of 4934) showing significant evolutionary relationships (using PGLM, threshold p = 0.01; seeMethodsfor details) between principal component (PC) axes and ecological/genomic traits of interest (for hypotheses, see Table **1**; for PC axis loadings, see Figure **1**)**

**Trait**	**Number of significant relationships**							
	**PC1**	**PC2**	**PC3**	**PC4**	**PC5**	**PC6**	**PC7**	**PC8**
Genome size	49	44	49	36	13	21	25	26
Intron percent	**651**	**561**	**493**	**539**^**+**^	**349**^**-**^	**313**^**+**^	**363**	**475**
Dimorphism	73	58	47	82	22	27	34	53
Thorax width	128	104	49	99	33	35	50	86
Specific development time	**393**	**309**	**272**	**267**^**+**^	170	161	197	**270**
Development time	118	86	31	87	31	33	37	79
Diet breadth	302*	229*	**200**^**-**^	**229**	119^+^	120	148^-^	**205**^**+**^
Ovariole number	**394**	**288**	**260**	**311**	162	179	195	**250**

Values over 200, or 4 × the expected number of proteins, we deemed significant and are given in bold. +/− in superscript indicates that regression coefficients are biased towards positive or negative values (binomial sign tests). Asterisk indicates the sign of coefficients are unevenly distributed among protein functional categories (χ^2^ tests).

**Figure 6:**
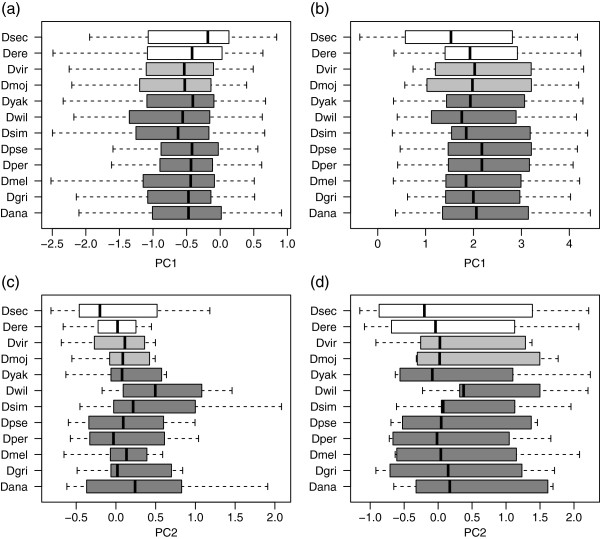
**Boxplot showing median ± IQR (box) and 95% CI (whiskers) of standing variation PC score (axes in Figure **1**) across 12 *Drosophila *species, for subset of proteins exhibiting a relationship with diet breadth: (a) PC1, Nucleic acid binding proteins (n = 26); (b) PC1, Transporters (n = 12); (c) PC2, Oxidoreductases (n = 9); (d) PC2, Transferases (n = 9).** Species are given in order of diet breadth: white bars, specialist; light grey bars, oligophagous; dark grey bars, generalist. Species names have been abbreviated using their first three letters.

For diet breadth (significant associations in 302 and 229 proteins across PC1 and PC2, respectively), the significant positive and negative associations were distributed unequally among protein functions (χ^2^ tests, p < 0.05). Among this subset of proteins showing significant associations, nucleic acid binding proteins and transcription factors showed predominantly positive associations between diet breadth and PC1, while transporters showed negative associations. For PC2, nucleic acid binding molecules and transcription factors showed positive associations, whilst oxidoreductases, transferases and select regulatory molecules showed mostly negative associations. Rather than being a phylogeny-wide trend, though, these patterns were driven by *D. sechellia*, the most resource-specialized of all the flies represented here and for whom this subset of proteins had diverged somewhat from the rest of the species (Figure 6). In *D. sechellia*, this subset of nucleic acid binding proteins and transcription factors had the highest PC1 (i.e. were most hydrophobic, with highest S and lowest N and O) and PC2 (i.e. the least polar, with the highest C), while its transporters had the lowest PC1 and its oxidoreductases, transferases and select regulatory molecules had the lowest PC2. Although these differences appeared to drive evolutionary correlations with diet breadth, from a stoichioproteomic perspective we might, *a priori,* have expected the protein composition of cactus-feeders such as *D. yakuba* to have been most distinctive (see e.g. [25]), owing to the low nutritional quality of their food resources, but this was not the case. Furthermore, *D. erecta* is almost as strictly specialized as *D. sechellia* but did not have distinctive stoichiometry in these proteins (Figure 6). It may therefore be different, species-specific selection pressures in *D. sechellia*, such as detoxification of host substances, that have contributed to this divergence; patterns of selection in this subset of proteins may warrant further research.

Ecological selection pressures evident at the proteomic level have been detected previously using comparative analyses across whole kingdoms (see e.g. [2,7,11]); the relatively few substantial findings we report here may also reflect a relatively short divergence period (compared to divergence among kingdoms), or that differences in the ecologies of *Drosophila* are not substantial or consistent enough to generate the selection pressures we predicted – although major differences in body composition reflect those seen among the flies’ respective substrates [34], these differences may not ramify into the proteins. Given the scope of the proteomic datasets, our overview-style analysis was also necessarily very broad and coarse-grained. More detailed research into the atomic content of specific proteins or protein groups using *GRASP* may be better able to reveal effects of nutritional limitation upon protein atomic content among *Drosophila* species.

### Protein expression levels in *D. melanogaster*

Highly expressed proteins (i.e. proteins that impose substantial nutrient demands upon a cell) should theoretically evolve to be nutrient poor [2,6] and, conversely, nutrient-rich proteins should be down-regulated in times of low nutrient availability [13,20]. To test this hypothesis, and to illustrate the ease with which the information in *GRASP* can be integrated with other publicly available resources, we asked how atomic composition, specifically N content, was related to protein expression (FlyAtlas, http://www.flyatlas.org[26]) across different tissue types in *D. melanogaster*.

Bragg & Wagner [3] outlined two hypotheses to account for how nutrient conservation in highly expressed proteins might come about. First, relief of nutrient limitation might arise mainly from changes in expression, with nutrient-rich proteins down-regulated and nutrient-poor proteins up-regulated. This scenario predicts a proteome-wide negative correlation between expression levels and content of the limiting nutrient. Second, specifically up-regulated proteins may have evolved to be nutrient-poor, resulting in a negative expression-nutrient content relationship only in up-regulated proteins [3,20].

Expression was bimodal in all tissues, the lower distribution corresponding to low- or rarely-expressed genes (see e.g. [35]). To test among the three alternatives (the two predictions outlined above, plus a null hypothesis of no negative association between nutrient content and expression), we conducted analyses for each tissue separately. Specifically, we conducted piecewise regression, allowing us to separate the low expression and high expression clusters at the most likely point (corresponding to a log_2_ abundance of 5.5; see Methods).

**Figure 7:**
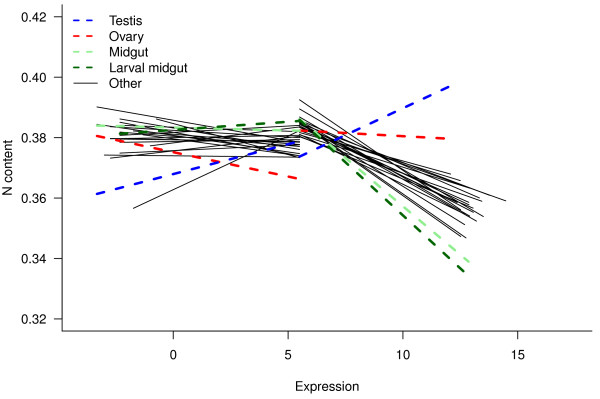
**Piecewise regression lines for N content against expression level (log**_**2 **_**transcript abundance) in 27 *D. melanogaster *tissues.**

Results of piecewise regressions for N content are shown in Table 3 (for context, statistical data for all tissues and all response variables [PC axes and all elements] are given in Additional file 1: Table S1). In the low expression cluster, N content was weakly and inconsistently related to expression levels. By contrast, in the high-expression cluster, N content was steeply negatively related to expression level in all tissues but the testes, where this relationship was actually positive, and ovaries, in which the slope did not differ from zero (Figure 7, Table 1).

**Table 3:** **Linear and piecewise regression statistics for models of N content against expression level (log**_**2**_**transcript abundance) in 27 *Drosophila melanogaster *tissues, in descending order of the estimated slope of the relationship in the high-expression cluster (expression > 5.5)**

	**Linear regression**		**Piecewise regression**				**Linear vs. piecewise**		
**Tissue**	**Estimate**	**SE**	**Estimate (< 5.5)**	**SE (< 5.5)**	**Estimate (> 5.5)**	**SE (> 5.5)**	**F**	**p**	**Sig.**
Larval midgut	−0.003092	0.000332	0.000539	0.001152	−0.007019	0.000798	21.74	<0.001	***
Adult midgut	−0.002606	0.000331	−0.000192	0.001157	−0.006411	0.000805	15.86	<0.001	***
Larval Malpighian tubule	−0.001542	0.000315	−0.000169	0.001168	−0.006314	0.000818	20.20	<0.001	***
Adult Malpighian tubule	−0.001756	0.000325	0.000103	0.001211	−0.006128	0.000803	18.41	<0.001	***
Larval hindgut	−0.002210	0.000324	−0.000267	0.001172	−0.005367	0.000821	10.35	<0.001	***
Adult hindgut	−0.002262	0.000330	−0.001324	0.001178	−0.005303	0.000799	8.81	<0.001	***
Cultured S2 cells	0.000309	0.000292	−0.000082	0.001210	−0.005190	0.000885	22.58	<0.001	***
Larval residual	−0.001548	0.000333	0.000140	0.001183	−0.004714	0.000812	10.04	<0.001	***
Larval fat body	0.000439	0.000355	0.003550	0.001219	−0.004449	0.000781	27.03	<0.001	***
Adult trachea	−0.000705	0.000323	0.000876	0.001216	−0.004427	0.000822	12.73	<0.001	***
Adult heart	−0.001064	0.000305	−0.000146	0.001105	−0.004321	0.000792	10.04	<0.001	***
Adult crop	−0.001076	0.000332	−0.000110	0.001232	−0.003892	0.000807	7.44	0.001	***
Whole adult fly	−0.001281	0.000401	−0.001290	0.001380	−0.003696	0.000883	4.94	0.007	**
Adult head	−0.002018	0.000349	0.000421	0.001221	−0.003671	0.000827	5.72	0.003	**
Mated spermatheca	−0.001020	0.000327	−0.000607	0.001188	−0.003608	0.000811	6.08	0.002	**
Virgin spermatheca	−0.000984	0.000322	0.000112	0.001154	−0.003364	0.000800	5.64	0.004	**
Larval salivary gland	−0.000248	0.000319	0.000352	0.001228	−0.003288	0.000814	8.24	0.000	***
Adult residual	−0.001764	0.000367	0.000961	0.001281	−0.003147	0.000785	5.19	0.006	**
Adult accessory gland	−0.000337	0.000337	−0.001018	0.001256	−0.002881	0.000836	6.46	0.002	**
Adult salivary gland	−0.000849	0.000361	−0.002055	0.001388	−0.002713	0.000774	4.99	0.007	**
Adult eye	−0.001458	0.000323	−0.000439	0.001152	−0.002687	0.000798	1.95	0.142	
Adult fat body	−0.001053	0.000341	−0.000622	0.001209	−0.002384	0.000791	1.75	0.174	
Thoracico-abdominal ganglion	−0.000649	0.000332	−0.001418	0.001237	−0.002359	0.000860	3.19	0.041	*
Larval CNS	0.000165	0.000323	−0.001832	0.001297	−0.002176	0.000937	7.43	0.001	***
Adult brain	−0.000537	0.000323	−0.001098	0.001241	−0.002089	0.000906	2.20	0.111	
Ovary	0.000965	0.000277	−0.001613	0.001319	−0.000418	0.001010	6.68	0.001	**
Testis	0.001925	0.000345	0.001978	0.001226	0.003557	0.000937	1.85	0.157	

For details, see Methods.

This indicates that, specifically in the highly expressed proteins of all tissues except the germline, increased expression was associated with conservation of N in protein sequences. In the testes, upregulated proteins were actually higher in N - the only tissue for which this was the case. The most steeply negative expression/N relationships were seen in the midguts of adults and larvae. In these tissues, doubling expression (i.e. increasing by one log_2_ unit) was associated with approx. 0.01 fewer N atoms per amino acid residue. The next-steepest relationships were also all gut-related tissues (hindgut and malpighian tubules; Table 3).

One clear interpretation of these patterns is that high levels of protein expression place a high demand for N upon somatic cells, creating a selection pressure for conservation of N in the most highly expressed proteins [4]. Thus, our results support the hypothesis that specifically up-regulated proteins have evolved to be nutrient-poor [3,20] in keeping with the idea that proteins evolve to reflect material costs of their production [1,4]. Among eukaryotes, this specific expression/N content relationship has so far only been identified in plants [eg., 2, 9] and is weaker or absent in animals, possibly owing to relaxed selection for efficiency of N usage in heterotrophs [2]. Proteins involved in nutrient assimilation show strong evolutionary conservation of the element they assimilate [1], so we would expect *a priori* to see the steepest relationships between N content and expression at the sites of N assimilation, such as the gut. Accordingly, midgut tissues, the main site of nutrient uptake, showed the steepest relationships of all tissues – followed by all other gut tissues in both larvae and adults (Table 3). In contrast, sites where protein synthesis is arrested or reduced, such as the testes, are not expected to show such a pattern. In contrast to the testes, the ovary grows during adult life [36], but we still found a relatively shallow relationship between PC1 and expression in ovaries, suggesting they may also be under reduced selection for N conservation. As a potential hypothesis for future study, conservation of N in eggs may impair offspring performance, constraining egg proteins to be nutritionally expensive. Consistent with this, dietary protein deficiency differentially affects female fertility rather than lifespan in *Drosophila*[22]. Brain and CNS tissues, while actively growing and differentiating in larvae and adults [37], also showed comparatively shallow N content/expression relationships (Table 3); we hypothesize that, because the CNS is highly charge-sensitive, the intrinsic correlation between N content and protein charge may reduce the scope for N conservation in nervous tissues. However, the apparently shallow expression/N content relationship in these tissues remains an open question.

Interestingly, Elser et al. [2] also used *D. melanogaster* as a reference model organism for heterotrophs, and used it as a baseline in the comparison with autotrophs. They found that N content in *Drosophila* followed a U-shaped curve that actually *increased* with expression intensity in the most highly expressed proteins (their Figure 1b). Examination of their figure reveals that this trend is influenced by two outliers (possibly ribosomal proteins, a group with unusually high N [10,13]); excluding these two outliers, the remainder of the points in their figure agree with our data because the non-outlier data in [2] follow a weakly negative trend. This result accords with the authors’ main conclusion, because this negative trend is indeed shallower than in the plants they analysed, lending weight to the idea that N conservation is indeed relaxed in animals.

Comparing the extent of N conservation we observed in *D. melanogaster* with the results obtained by Elser et al. [2], our results indicate that it is important to consider tissue-specific expression levels. For example, in the tissues with the strongest N conservation, the larval and adult midgut, the most highly expressed proteins were approx. 0.05 N atoms poorer per residue than in the least highly expressed. By contrast, in the testes there was no such pattern. These results suggest that selection for nutrient conservation in proteins may be mediated by tissue-specific expression, a possibility that requires further research.

Of course, it is difficult to be entirely confident that stoichioproteomic patterns are not a result of systematic selection on biochemical properties of amino acids or of underlying DNA rather than elemental content *per se*. As an alternative hypothesis, the most highly expressed proteins may require a lower charge density to allow unbinding from the machinery of translation at a fast enough rate to maintain high expression, which would explain their lower N and O content, although this requirement would most likely be of much lower importance than requirements of protein function. Future authors may wish to make preliminary steps towards elucidating these two hypotheses by conducting analyses of protein composition and expression while controlling for charge density.

## Conclusions

We have provided a mainly descriptive account of broad-scale variation in the atomic content of *Drosophila* proteins across the 12 fully sequenced *Drosophila* species, to which *GRASP* provides ready access, alongside preliminary tests of some core stoichioproteomic hypotheses. Further detailed research using *GRASP* will provide deeper insights into the evolution of atomic composition within and among species. Subsequent releases of *GRASP* will be augmented with similar information on other organisms across the phylogeny, as well as with additional information about other characteristics, including known developmental regulators, life span, feeding habits, and other ecological information, resulting in a powerful bioinformatics knowledgebase for the framing and testing of stoichioproteomic hypotheses.

We found that atomic content in *Drosophila* was at least partially a function of DNA GC content and amino acid biochemistry, and was also predictable based upon relative amounts of other constituent elements. On top of this, however, proteins carried signatures of conservation of limiting nutrients: N content was reduced in the most highly expressed proteins in most somatic tissues, but not in testes where nutrient conservation is unnecessary. However, the predictable patterns in elemental composition that we detected within proteomes were not plainly evident in broad-scale comparisons across species, indicating a potential role for lineage-specific evolutionary changes; this phylogenetic variation can provide a testing ground for future researchers wishing to use *GRASP* to look into the evolution of atomic composition.

Protein atomic content can be seen a passive emergent property of selection acting on the phenotype via a protein’s structure, but may also be a source of selection pressure in itself, through its effect on organism nutrient demand. Here we have identified patterns in atomic content ranging from associations with basic properties of DNA to evolutionary associations with ecological species differences that may represent signatures of selection for nutrient conservation. We hope that the stoichioproteomic trends we have identified here will provide multiple working hypotheses for future research aiming to investigate these hypotheses in detail using these 12 *Drosophila* species and beyond. *GRASP* will provide a convenient springboard for such studies.

## Methods

*GRASP* is organized around a central interface whereby users select the species they wish to query and then the category of proteins whose data they wish to extract. A range of data is included on the website to enable direct tests of hypotheses, as well as providing links to outside sources of information. We have added categorical data mapping to the Gene Ontology (http://www.geneontology.org) on protein family, biological process, molecular function, and *pathway*, derived from FlyBase (http://www.flybase.org), Panther (http://www.pantherdb.org), and Uniprot (http://www.pir.uniprot.org). *GRASP* also includes the amino acid sequence itself, along with its length, plus the underlying coding DNA sequence and information about its GC content, a property that directly affects the amino acid sequence [14]. The current sequence data in *GRASP* are derived from FlyBase version FB_2007_3, October 2007. When a gene gives rise to more than one protein product, each protein product is indicated with a different suffix (i.e., PA, PB, etc.). Aggregations grouped by biological process, molecular function, protein pathway and family can be selected and output to the browser or *via* downloadable spreadsheets and comma-separated value lists for use in other statistical software. In addition, users can create their own aggregations of proteins derived from the selected species, automatically generating a downloadable spreadsheet of aggregated amino acid and elemental counts.

### Exploratory analysis of *Drosophila* proteomes

After downloading the information from *GRASP*, all data analyses were carried out in R 2.13.0 [38] using various packages as cited below. Unless stated otherwise, only proteins with orthologs in all 12 species were analysed.

First, we used principal component analysis to characterize multivariate relationships between C, O, N and S content, DNA GC content, protein length, and the proportions of hydrophobic, polar, positive, negative and aromatic residues, respectively, in the entire dataset. We then asked whether proteins from the 12 different species, and different functional categories, occupied distinct regions in multivariate space using MANOVA with the first 8 principal components as a multivariate response.

### Species- and clade-specific divergence in elemental content

**Figure 8:**
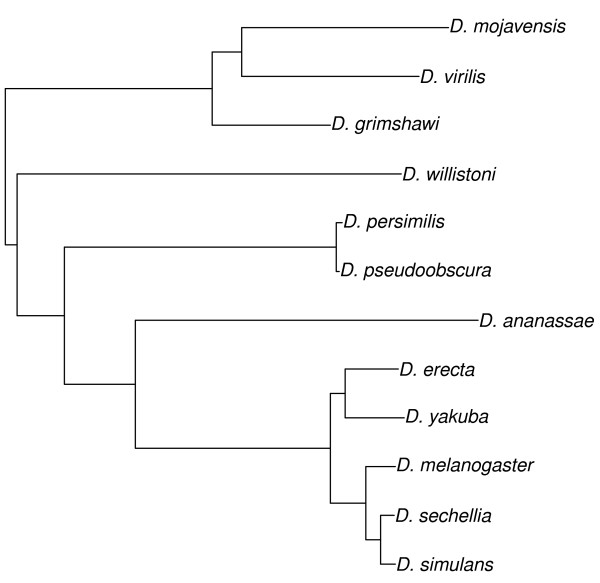
**Phylogeny of the 12 *Drosophila *species used in this study.** (Taken from [17]).

Because species share evolutionary history to different extents, comparisons among species must account for the way characters evolve e.g. [39-41]. For the 12 *Drosophila* species under consideration, a well-supported phylogeny is known based on the whole genome, with reliable divergence estimates (Figure 8; see [17]). Both the inferred phylogeny and protein atomic content are closely linked to the amino acid sequence, which may lead to circular inference – we assumed this would not bias our results, i.e. we assumed that forces affecting the atomic composition of proteins were independent of the forces affecting the sequence affinity of the entire genomes on which the phylogeny was based.

First, we looked at species divergence in the PC axes identified above by reconstructing the ancestral states for each protein across the phylogeny on each axis in the PCA using maximum likelihood reconstruction in the ace() function of the ape package in R [42]. We used this information to calculate the estimated divergence for each protein in each species since its most recent common ancestor with a sibling species. Lineage- and clade-specific evolution of atomic content could therefore be isolated from patterns shared among species.

### Evolutionary patterns in elemental content and ecology

We used the method of phylogenetically independent contrasts (PIC, [39]) to calculate independent contrasts in all considered variables. To look at multivariate evolutionary change we then calculated principal components in these contrasts, following [33]. Evolutionary associations among the variables were assessed using the variable loadings of the principal axes.

### Testing hypotheses across the phylogeny

To test ecological and genomic hypotheses relating to stoichioproteomics (Table 1), we asked whether any of the species-level ecological or genomic traits listed in Table 1 on its own was related systematically to evolutionary patterns in atomic composition and protein properties. We used phylogenetic generalized least squares (PGLM), using the CAIC package [43] to model phylogenetic changes in the principal component axes identified above for “standing variation” against changes in the trait of interest (i.e. for each ecological trait, 4934 analyses each of n = 12), asking whether fitted lines systematically departed from zero. Under a null hypothesis we would expect 1% of 4934, or 49, analyses to be significant at the 0.01 level; we used 200 or approx. 4 times this number as an arbitrary but conservative threshold for significance. Note that PGLM differs from the method of PIC which we used to calculate the EPC axes: where PIC calculates a new dataset of phylogenetically independent contrasts, PGLM instead uses raw species values as the response variable, and incorporates phylogenetic information into the error term of the model. Thus, we performed these analyses on the "standing variation" PC axes (rather than the EPC axes). For ecological variables that were frequently associated with protein composition (intron percent, ovariole number, specific development time and diet breadth) we asked whether associations were consistently positive or negative in particular protein categories using χ^2^ tests; for each variable, Table 1 outlines hypotheses relating to specific subsets of proteins that might be expected to show elemental conservation in their sequences.

### Protein expression in *D. melanogaster*

Detailed information on protein expression in *D. melanogaster* has recently become available in the FlyAtlas database (http://www.flyatlas.org[26]). We used FlyAtlas to analyse protein elemental content with respect to protein expression in various tissues of *D. melanogaster* (see Table 3 for tissues). Nutrient conservation in proteins is expected to appear as a negative relationship between protein nutrient content and protein expression level (see [2,3,13]). If N conservation is brought about by wholesale adjustment of expression levels on the basis of N content, we would expect to see such a negative relationship across all proteins. On the other hand, if proteins that are constrained to be highly expressed have evolved to be low in N, we should see this negative relationship only in highly-expressed proteins [3].

To test between these two hypotheses, we fitted piecewise regression models to the data for each tissue, breaking the bimodal distribution at a point corresponding to a log_2_ abundance of 5.5 (determined by comparing AIC values of piecewise regressions using different breakpoints; data not shown).

In tissues where N conservation is expected to be weak or non-existent, however, we would expect a negative relationship in neither down- nor up-regulated proteins. Thus, we predicted that the slope of any relationship between expression and N content would be shallower for the testes than for any other tissue.

## Competing interests

The authors declare no competing interests, financial or otherwise.

## Authors’ contributions

JDJG carried out the analyses and drafted the manuscript. CA participated in the design and implementation of *GRASP*, in the analyses and in drafting the manuscript. HMM participated in the analyses and in drafting the manuscript. JJE, SK and WFF conceived of the study, implemented and currently maintain *GRASP* and provided comments on the manuscript. All authors read and approved the final manuscript.

## Supplementary Material

Additional file 1: Table S1 Results of linear and piecewise regression of different response variables (PC1-4 and C, O, N and S content) against expression level for 27 different *Drosophila melanogaster* tissues. See text for details. Click here for file
